# Human herpesvirus 7 and the risk of developing multiple sclerosis

**DOI:** 10.1093/braincomms/fcaf492

**Published:** 2025-12-18

**Authors:** Jens Ingvarsson, Viktor Grut, Rasmus Gustafsson, Martin Biström, Lea Lambert, Birgitta E Michels, Tomas Bergström, Louis Flamand, Tim Waterboer, Peter Sundström

**Affiliations:** Department of Clinical Sciences, Neurosciences, Umeå University, Umeå 90187, Sweden; Department of Clinical Sciences, Neurosciences, Umeå University, Umeå 90187, Sweden; Department of Clinical Neuroscience, Karolinska Institutet, Stockholm 17177, Sweden; Department of Clinical Sciences, Neurosciences, Umeå University, Umeå 90187, Sweden; Department of Microbiology, Infectious Disease and Immunology, Faculty of Medicine, Laval University, Quebec City, QC G1V 0A6, Canada; Department of Infectious and Immune Diseases, CHU de Québec Research Center, Laval University, Québec, QC G1V 4G2, Canada; Infections and Cancer Epidemiology, German Cancer Research Center, Heidelberg 69120, Germany; Department of Infectious Diseases, Institute of Biomedicine, Sahlgrenska Academy, University of Gothenburg, Gothenburg 40530, Sweden; Department of Microbiology, Infectious Disease and Immunology, Faculty of Medicine, Laval University, Quebec City, QC G1V 0A6, Canada; Department of Infectious and Immune Diseases, CHU de Québec Research Center, Laval University, Québec, QC G1V 4G2, Canada; Infections and Cancer Epidemiology, German Cancer Research Center, Heidelberg 69120, Germany; Department of Clinical Sciences, Neurosciences, Umeå University, Umeå 90187, Sweden

**Keywords:** multiple sclerosis, risk factors, serology, human herpesvirus 7, Epstein–Barr virus

## Abstract

Epstein–Barr virus is now regarded as the critical risk factor for multiple sclerosis. However, Cytomegalovirus and human herpesvirus 6A have also been associated with altered multiple sclerosis risk, suggesting a multifactorial aetiology. Here, we present the first large-scale study of the association between human herpesvirus 7 and the risk of developing multiple sclerosis. A nested case-control study was performed by crosslinking Swedish registries and biobanks, identifying blood samples from 981 cases who later developed multiple sclerosis and 1278 matched controls. Serological testing was performed with a multiplex immunoassay. The association between viral serostatus and the risk of developing multiple sclerosis was analysed with conditional logistic regression, calculating an odds ratio with 95% confidence interval. Interactions between antibodies against human herpesvirus 7 and the Epstein–Barr virus nuclear antigen 1 regarding multiple sclerosis risk were analysed on the additive scale. Serological evidence of human herpesvirus 7 infection was associated with a higher risk of developing multiple sclerosis: odds ratio = 2.2 (95% confidence interval = 1.8–2.7), *P* < 0.001. The results remained similar when adjusting for cytomegalovirus, Epstein–Barr virus and human herpesvirus 6A serostatus. Synergistic interactions between human herpesvirus 7 and Epstein–Barr virus nuclear antigen 1 seroreactivity were observed: attributable proportion due to interaction = 0.51 (95% confidence interval = 0.34–0.68). These results suggest that human herpesvirus 7 could be a contributing factor in multiple sclerosis aetiology.

## Teaser

Ingvarsson *et al*. report that serological evidence of infection with human herpes virus 7 is associated with a higher risk of developing multiple sclerosis. They also report a synergistic interaction between human herpes virus 7 and Epstein–Barr virus regarding the risk of developing multiple sclerosis, suggesting a mechanistic link.

## Graphical Abstract

**Graphical Abstract:**
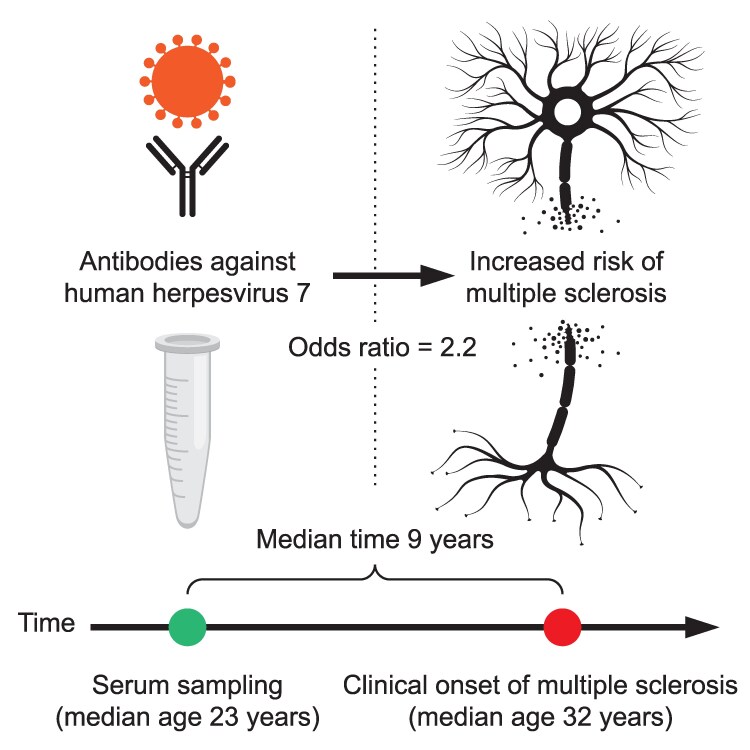

## Introduction

While multiple sclerosis (MS) has been associated with several viruses, the most robust association is with Epstein–Barr virus (EBV).^1^ Studies have shown that EBV infection appears to be a prerequisite for MS development.^2^ In addition, the analysis of samples collected before clinical onset of MS has shown that antibodies against Human Herpesvirus 6 (HHV-6),^2^ HHV-6A^3,4^ and rubella virus^5^ also are associated with an increased risk of developing MS, while antibodies against cytomegalovirus (CMV)^6^ are associated with a decreased risk of MS.

All of these viruses, except for rubella, belong to the *Herpesviridae* family, where HHV-6A and CMV belong to the *beta-Herpesvirinae* subfamily, in which also HHV-6B and HHV-7 are members. Being genetically similar, it was not until 2012 that HHV-6A and 6B were classified as two distinct viruses and a validated method for differentiating them serologically is still lacking. In 1990, HHV-7 was first isolated from CD4+ T-lymphocytes.^7^

Like many other members of the HHV family, HHV-7 is shed in saliva, constituting a major transmission route.^8^ Infection with HHV-7 normally occurs in early childhood, on a group level somewhat after HHV-6 infection,^9^ and results in life-long latent infection.^10^ A 97% seroprevalence of HHV-7 was initially reported (28 out of 29 adults),^11^ but later estimates on much larger samples have shown seroprevalences of 35% to 85%.^12,13^ Skin disorders like exanthema subitum (roseola) and pityriasis rosea, as well as febrile seizures, are suspected associated diseases. Rare cases of encephalitis associated with HHV-7 have been reported, suggesting a neurotropic potential of HHV-7.^10^

Until now, there has been almost no support for an association between HHV-7 and MS, and investigations are both sparse and contradictive. On the one hand, a study of the presence of HHV-7 DNA, mRNA and protein in adult MS brain tissue only found low levels of virus expression, and did not support the role of HHV-7 in MS.^14^ Furthermore, a 2023 meta-analysis on HHV and MS concluded that there was no significant association between HHV-7 and MS risk.^15^ On the other hand, HHV-7 DNA was detected in the blood of 33% of MS cases compared to 10% of controls in a Tunisian study.^16^ A recent Finnish study also reported that people with MS had higher viral reads of EBV, HHV-6B and HHV-7 in deep cervical lymph nodes.^17^

In our earlier pre-symptomatic studies, we have found that elevated antibodies against EBV and HHV-6A were associated with an increased risk for MS. No such association was seen for HHV-6B. The aim of the present study was to study if the serological response to HHV-7 is associated with MS risk in a larger sample.

## Materials and methods

### Study population

In this nested case-control study, bio-banked plasma or serum samples from individuals that later developed relapsing-remitting MS (RRMS) and matched controls were included. Participants were born in the years 1937–2000. This material is an extension on a previous material, collected using the same method of recruitment and case verification as previously described.^18^ Briefly, cases were identified in the Swedish national MS registry and the earliest available Swedish bio-banked sample, collected before onset of RRMS and before age 40 (previous material, ‘2012 cohort’) or age 25 (extension material, ‘2020 cohort’), was included. For each case, controls were included from the same biobank, matched by sex, sampling date, and date of birth. In contrast to the one-to-one matching used in the 2012 cohort, the additional cases in the 2020 cohort included in this study were matched to 1–2 controls. A detailed description of this process is presented in the Supplementary material (Supplementary Fig. 1).

### Laboratory procedures

A previously described and validated bead-based multiplex assay^19^ was used to perform serological analyses on all samples. The analysis, performed at the German Cancer Research Center (DKFZ), Heidelberg, Germany, included serologies for antigens on EBV, CMV, HHV-6A and HHV-7. Antigen reactivity was measured as median fluorescent intensity (MFI). For EBV and CMV, previously defined and validated cut-offs defining seropositivity were used (Supplementary Table 1).^19^ For HHV-6A and HHV-7, there are no validated reference methods to define seropositivity. As in previous studies on HHV-6A, the 75th percentile of reactivity towards the HHV-6A immediate-early 1 antigen (IE1A) in the controls (MFI 150.25) was thus used as a cut-off to define high seroreactivity.^4,20^ Using the visual inflection point (VIP) method,^21^ a cut-off at MFI 150 for the IE1A antigen was suggested, supporting our 75th percentile cut-off. For HHV-7, the VIP method was also used to determine the cut-off for seropositivity towards the HHV-7 antigen U14, set at MFI 225. The U14 assay has been validated against an external assay (Supplementary material, development and validation of a high through-put serological assay for HHV-7; Supplementary Fig. 4). For a subset of the participants, HLA data were retrieved from previous Swedish studies or from PCR analyses of saliva samples, as described previously.^5^

### Statistical analyses

Viral seroreactivity in cases and controls was compared with Mann–Whitney U-test. The association between HHV-7 seropositivity and the risk of developing MS was analysed with conditional logistic regression, calculating odds ratios (OR) with 95% confidence interval (CI). The analyses were adjusted for CMV, EBV and HHV-6A serostatus. Since EBV seropositivity is associated with MS in opposite directions depending on age at sampling, the analyses were stratified by sampling age <20 or ≥20 years.^22^ Matched sets with discordant age categories were assigned to the strata of the case. To test if the association between HHV-7 and MS risk has a dose-response relationship, we used quintiles of HHV-7 U14 seroreactivity in a conditional logistic regression.

Sensitivity analyses were performed in samples drawn >8 years^6^ before the clinical onset of MS, and in the subset of samples with available data on HLA-DRB1*15 and HLA-A*02 carriership.

Interactions between HHV-7 seropositivity and the EBV nuclear antigen 1 (EBNA-1) seroreactivity regarding the risk of developing MS were analysed on the additive scale. EBNA-1 seroreactivity was dichotomized as high or low, based on the median MFI in controls, as in previous studies.^4,20^ Attributable proportion due to interaction (AP) was calculated with conditional logistic regression. CIs for AP were calculated with the delta method. The exposure with the lowest risk of MS was used as reference.

Statistical tests were two-tailed. The significance level was 0.05. Analyses were performed in SPSS, version 28.0.1 and R, version 4.4.2.

### Ethical statement

The Regional Ethical Review Board in Umeå approved the project (2011-198-31M with subsequent amendments). The study was performed in accordance with the Declaration of Helsinki. All participants were informed via letter and had the possibility to opt-out. All individuals who participated in the genetic analyses signed written informed consent forms.

## Results

The analysis generated results for a total of 981 cases and 1278 matched controls (Supplementary Fig. 1). Median age at sampling was 22.2 years (inter-quartile range 19.0–25.6) and median age at MS onset was 32.1 years (inter-quartile range 27.0–38.3, Table 1; for individual participants see Supplementary Fig. 2). The mean within-set absolute difference in sampling date was 4.8 days, and absolute mean within-set difference in birth date was 3.7 months.

**Table 1:** Characteristics of cases and controls

	Cases	Controls
Total *n*	981	1278
Sex (male/female, *n*)	201/780	277/1001
Age at sampling (years)	22.6 (19.3–26.6)	21.9 (18.8–25.0)
Time to MS onset (years)	8.9 (4.1–14.4)	–
HHV-7+	81.7%	68.0%
CMV+	50.1%	58.3%
EBV+	86.2%	83.3%
HHV-6A+	34.3%	25.0%

Median (25th–75th percentile) for continuous variables. HHV-7+, seropositive for human herpesvirus 7; CMV+, seropositive for cytomegalovirus; EBV+, seropositive for Epstein–Barr virus; HHV-6A+, seropositive for human herpesvirus 6A. Definitions and cut-offs for seropositivity and antigens for each virus are available in Supplementary Table 1.

The median seroreactivity against HHV-7 was higher in cases than in controls, 422 vs 338 MFI, *P* < 0.001 (Supplementary Fig. 3). Seropositivity against HHV-7 was associated with an increased risk of developing MS, OR = 2.2 (95% CI = 1.8–2.7), *P* < 0.001. Similar results were observed when the 2012 and 2020 cohorts were analysed separately, and when stratifying for sampling age (Supplementary Table 2). The results also remained similar in multivariable analyses, adjusting for EBV, HHV-6A and CMV serostatus (Table 2). Higher HHV-7 seroreactivity was associated with a higher risk of developing MS (Table 3).

**Table 2:** HHV-7 serostatus as risk factor for developing MS

	Cases, *n* (%)	Controls, *n* (%)	OR	95% CI	*P*
All ages, *n* = 981 + 1278					
HHV-7+	801 (82%)	869 (68%)	2.08	1.66–2.59	<0.001
CMV+	491 (50%)	745 (58%)	0.7	0.58–0.84	<0.001
EBV+	846 (86%)	1064 (83%)	1.12	0.86–1.47	0.41
HHV-6A+	336 (34%)	320 (25%)	1.44	1.17–1.76	<0.001
Age <20, *n* = 291 + 431					
HHV-7+	223 (77%)	262 (61%)	2.38	1.65–3.45	<0.001
CMV+	131 (45%)	212 (49%)	0.86	0.63–1.19	0.37
EBV+	189 (65%)	307 (71%)	0.65	0.45–0.94	0.02
HHV-6A+	78 (27%)	105 (24%)	0.99	0.69–1.42	0.93
Age ≥ 20, *n* = 690 + 847					
HHV-7+	578 (84%)	607 (72%)	1.89	1.42–2.50	<0.001
CMV+	360 (52%)	533 (63%)	0.61	0.48–0.76	<0.001
EBV+	657 (95%)	757 (89%)	2.29	1.47–3.57	<0.001
HHV-6A+	258 (37%)	215 (25%)	1.68	1.31–2.16	<0.001

OR, odds ratio; CI, confidence interval; HHV-7+, seropositive for human herpesvirus 7; CMV+, seropositive for cytomegalovirus; EBV+, seropositive for Epstein–Barr virus; HHV-6A+, high seroreactivity for human herpesvirus 6A. Definitions of seropositivity and cut-offs for each viral antigen are provided in Supplementary Table 1.

**Table 3:** Quantitative effect of HHV-7 seroreactivity on the risk of developing MS

	Quintiles of HHV-7 antigen U14 (MFI)					
Sampling age	1 (ref)	2	3	4	5	*P* trend^a^
All ages	1.00	1.63 (1.17–2.27)	2.40 (1.76–3.26)	2.34 (1.71–3.21)	2.93 (2.12–4.05)	<0.001
All ages, adj^b^	1.00	1.58 (1.13–2.21)	2.32 (1.70–3.18)	2.16 (1.57–2.97)	2.63 (1.89–3.65)	<0.001
Age <20 years	1.00	1.91 (1.10–3.31)	2.42 (1.43–4.09)	3.35 (1.94–5.80)	4.29 (2.39–7.69)	<0.001
Age <20 years, adj^b^	1.00	2.01 (1.15–3.53)	2.56 (1.50–4.35)	3.51 (2.00–6.16)	4.44 (2.46–8.03)	<0.001
Age >20 years	1.00	1.48 (0.98–2.24)	2.28 (1.55–3.35)	1.95 (1.32–2.89)	2.46 (1.65–3.65)	<0.001
Age >20 years, adj^b^	1.00	1.35 (0.88–2.08)	2.04 (1.37–3.04)	1.65 (1.10–2.47)	1.93 (1.28–2.92)	<0.01

Figures represent odds ratio (95% CIs). Limits for quintiles: 167; 273; 421; 646.6 MFI, derived from the distribution in controls.

^a^Calculated by analysing quintiles as continuous variables in conditional logistic regression.

^b^Adjusted for CMV, EBV and HHV-6A serostatus, as defined in Supplementary Table 1.

The sensitivity analysis of samples with more than 8 years to the clinical onset of MS (*n* = 530 cases and 712 controls) remained statistically significant for HHV-7: OR = 2.0 (95% CI 1.5–2.6), *P* < 0.001. The association between HHV-7 and a higher risk of developing MS also remained significant when adjusting for HLA-DRB1*15 and HLA-A*02 genotype (Supplementary Table 3).

**Figure 1:**
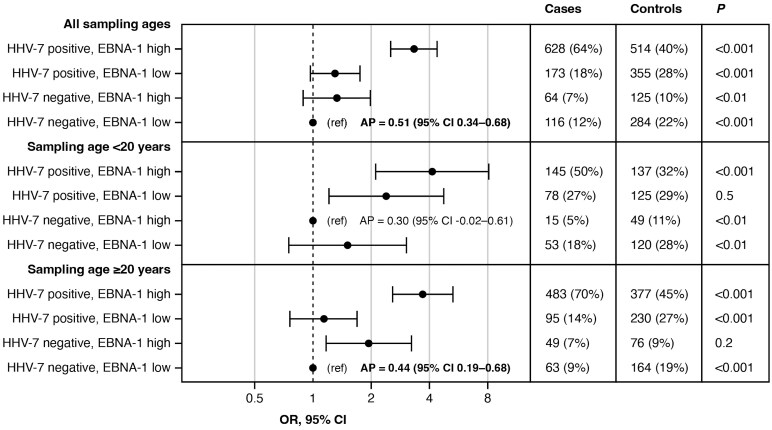
Interactions between HHV-7 seropositivity and EBNA-1 seroreactivity regarding the risk of developing MS. ORs are presented on Log2 scale. HHV-7 positive, Human herpesvirus 7 antigen U14 ≥ 225 MFI; EBNA-1 high, seroreactivity against Epstein Barr virus antigen EBNA-1 > median MFI in controls (4571.5 MFI); OR, odds ratio; CI, confidence intervals; AP, attributable proportion due to interaction.

A significant synergistic interaction was observed between HHV-7 seropositivity and high EBNA-1 seroreactivity. This interaction was observed in the whole sample and in the older strata (Fig. 1).

## Discussion

The cumulative body of data that has been generated by the scientific community over the years currently suggests that no singular virus is sufficient to cause MS. While the strongest association to MS has been seen for EBV, MS is also associated with other viruses. Research from our group using serological analyses on pre-symptomatic samples has highlighted HHV-6A, CMV and EBV from the *Herpesviridae* family, and the present work adds HHV-7 as a possible risk factor for MS. We have also shown statistical interactions between CMV and EBV, CMV and HHV-6A, EBV and HHV-6A, and in the present study between EBV EBNA-1 seroreactivity and HHV-7 seropositivity, suggesting that all of these interact biologically regarding risk for MS development.^6,20^ Similar to HHV-6A, HHV-7 may act in concert with EBV. While HHV-6A and HHV-7 are different viruses, they are largely similar from a genetic viewpoint and probably also share pathogenetic features. It is therefore possible that HHV-6A and HHV-7 infection have similar effects on MS risk. Despite the genetic similarities, the gene encoding the IE1 antigen is sufficiently divergent to discriminate between antibodies to HHV-6A and HHV-6B,^4^ why cross-reactivity to the more distantly related HHV-7 antigen is not anticipated.

Recently, a Japanese study of endogenous (inherited chromosomally integrated) HHV-6B showed an association with systemic lupus erythematosus, and an increased risk for other autoimmune diseases was suggested.^23^ In the same study, this finding was replicated in a population with European ancestry. Endogenous HHV-6 is present in ∼1% of humans, but the prevalence in people with MS is unknown. Whether HHV-7 also may establish latent infection through chromosomal integration is currently under investigation.

The interaction between antibodies against HHV-6A and EBV regarding risk for MS could hypothetically result from the ability of HHV-6A to reactivate latent EBV.^20,24^ Infection with HHV-7 has been reported both with simultaneous primary EBV infection and reactivation.^25^ Both HHV-6A and HHV-7 could thus constitute a possible second hit, amplifying the aberrant effect of EBV on the immune system and thereby contributing to MS development. Possibly, simultaneous infection with more than one herpesvirus is not a rare event and could lead to more pronounced symptoms of infection or altered effects on the immune system. Since herpesviruses are ubiquitous and often transmitted through an efficient saliva route, this could well be a common scenario.

The similarity between symptoms from relapsing-remitting MS and those from latent herpesvirus infection, which may reactivate and produce flares of symptoms, has been noted for several decades. However, the central role of EBV in MS aetiopathogenesis has only recently been convincingly shown.^1^ Most interestingly, EBV resides in B-lymphocytes that constitute key players in MS pathology. The central role of B-cells in MS has been demonstrated through the effectiveness of anti-CD20 therapies, targeting B-lymphocytes exclusively.^26^ It is therefore thought-provoking that the other key component in immune mediated diseases, the CD4+ T-lymphocyte, is where HHV-7 resides.^10^

In addition, HHV-7 may have neuropathogenic potential. In addition to encephalitis, several central nervous system disorders such as meningitis and myelitis have been described during HHV-7 infection, and HHV-7 DNA or protein has been found in meninges, oligodendrocytes and neurons.^10^

For EBV, age at infection appears crucial: infection after adolescence is associated with a several-fold risk increase for MS.^22^ This is mirrored by the often asymptomatic EBV infection in children, while EBV infection in adulthood often results in infectious mononucleosis. Similar observations have been made for HHV-7, however with a different time frame. When HHV-7 infection was delayed into adolescence, neurological diseases such as encephalitis were more common.^27^

If infection with HHV-7 is involved in MS aetiopathogenesis, some treatment opportunities may emerge. We are not aware of any vaccine development programmes, but antiviral treatment (acyclovir) was shown to be more effective than follow-up in Pityriasis rosea.^28^ Interestingly, a randomized double-blind study on valacyclovir has been performed in MS. The rational for that study did not specifically involve HHV-7 and the study on 70 MS patients was negative regarding the primary endpoint. However, a subgroup with highly inflammatory active disease did show an effect on new active lesions associated with antiviral treatment.^29^

Strength with the present study is the sample size with robust findings of associations between HHV-7 antibodies and MS risk in both the 2012 cohort and the 2020 cohort. The results also remained significant in sensitivity analyses in samples drawn more than 8 years before MS onset, arguing against reverse causation. Likewise, the association between HHV-7 and MS risk remained significant in the subset of samples with available HLA data. This finding argues against HLA confounding, such as preferential presentation of the U14 antigen by HLA-DRB1*15.

However, it should be acknowledged that for each participant, we have only retrieved the first available serum sample in the biobank. To assess the trajectory of seroreactivity over time on the individual level, future studies should include repeated consecutive samples. Furthermore, there is currently no validated reference method to define HHV-7 seropositivity. Although the serological assay has been validated against an external method (Supplementary material), there is yet no available reference panel. The cut-off for defining HHV-7 seropositivity can thus not be validated. Still, it should be noted that we also observed an association between higher levels of HHV-7 seroreactivity and a higher risk of MS. This quantitative effect is not dependant of a cut-off for seropositivity. The sparsity of other studies on this topic calls for further exploration of the association between HHV-7 and MS.

In conclusion, serological evidence of previous HHV-7 infection was associated with a higher risk of developing MS. Our findings also indicate synergy between HHV-7 and EBV regarding MS risk. We therefore suggest further studies on the link between HHV-7 and MS.

## Supplementary Material

fcaf492_Supplementary_Data

## Data Availability

The data that support these findings are available from the corresponding author on reasonable request.
