# Supplementary material for: Human herpesvirus 7 and the risk of developing multiple sclerosis
Source: Brain Commun. 2025 Dec 18;8(1):fcaf492. doi: 10.1093/braincomms/fcaf492 (PMC12782018; doi:10.1093/braincomms/fcaf492)
Supplement: fcaf492_Supplementary_Data [file fcaf492_supplementary_data.pdf]

Supplementary material for

Human herpesvirus 7 and the risk of developing multiple sclerosis

Contents:

- **Methods and materials**
- **Supplementary Figure 1**
- **Supplementary Table 1**
- **Supplementary Figure 2**
- **Supplementary Figure 3**
- **Supplementary Table 2**
- **Supplementary Table 3**
- **Development and validation of a high through-put serological assay for HHV-7**
- **Supplementary Figure 4**

Methods and materials

The Swedish MS registry and a local MS database at Umeå University Hospital were used to identify individuals with MS. For each individual in these registries, the Swedish personal identification number and the date of MS onset were obtained. If the date of MS onset was missing in the registry, the date was retrieved from the medical records. If no such date could be obtained, the individual was excluded.

This information was next crosslinked with the registries at seven Swedish biobanks, containing remnants from clinical tests. By combining the personal identification numbers, the sampling dates, and the dates of MS onset, serum or plasma samples collected before the clinical onset of MS could be identified and retrieved from the biobanks.

This process was performed in two stages, 2012 and 2020 (supplementary figure S1). In the 2012 cohort, the earliest available samples collected before the clinical onset of MS and before the age of 40 were included. In the 2020 cohort, the age at sampling was limited to before 25 years of age. As in the 2012 cohort, the earliest available samples were included.

In the 2012 cohort, one control was included for each case, matched by sex, sampling date, and date of birth. Absolute matching was used for biobank and sex. Best match approach was used for sampling date and date of birth, prioritizing a low difference in sampling date to account for possible confounding by seasonal effects.

In the 2020 cohort, two controls were initially selected for each case. However, some of the selected controls could not be included in the study due to missing samples or insufficient sample volume. A few controls also declined to participate in the study. Some cases in the 2020 cohort were thus only matched with one control. Absolute matching was still used for sex and biobank, but the method to select controls based on sampling date and birth date was refined. The absolute difference between the case and the available controls was calculated for sampling date (in days) and birth date (in months) and added together. The two controls with the lowest sum of absolute differences were then selected for inclusion.

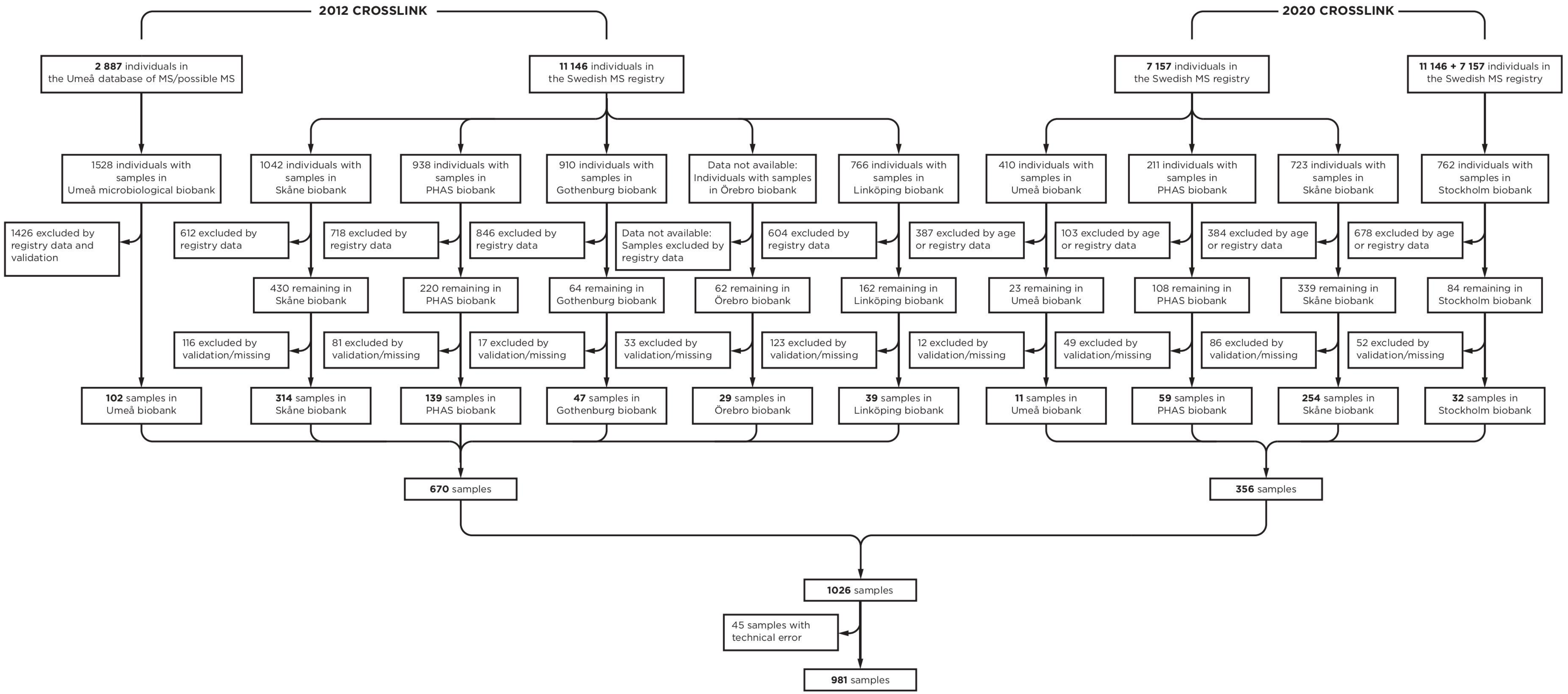

Supplementary Figure 1. Case ascertainment. Samples were identified through two separate crosslinks between the MS registry and the biobank registries, in 2012 and 2020. PHAS, Public Health Agency of Sweden.

Supplementary Table 1. Definitions of viral seropositivity

Virus	Antigen	Cut-off (MFI)	Definition of seropositivity
HHV-7	U14	225	Exceeding MFI cut-off
CMV	pp28	200	≥2 antigens exceeding MFI cut-off
	pp52	1101	
	pp150 N-terminus	655	
EBV	EBNA-1 trunc	1800	≥2 antigens (EBNA-1 trunc *OR* EBNA-1 pep) exceeding MFI cut-off
	EBNA-1 pep	600	
	VCA p18	2526	
	ZEBRA	200	
	EA-D	300	
HHV-6A	IE1A	150	Exceeding MFI cut-off

Note: MFI, median fluorescence intensity; HHV-7, human herpesvirus 7; CMV, Cytomegalovirus; Pp28, amino acids (aa) 1–189; pp52, aa 1–432; pp150 N-terminus, aa 1–550; EBV, Epstein Barr virus; EBNA-1 trunc, EBV nuclear antigen 1 truncated, aa 325–641; EBNA-1 pep, EBV nuclear antigen 1 peptide, aa 385–420; VCA p18, viral capsid antigen p18, aa 1–175; ZEBRA, Z-Epstein-Barr virus replication activator, aa 1–244; EA-D, early antigen-diffuse, aa 1–403; HHV-6A, human herpesvirus 6A; IE1A, truncated immediate-early protein 1 from HHV-6A.

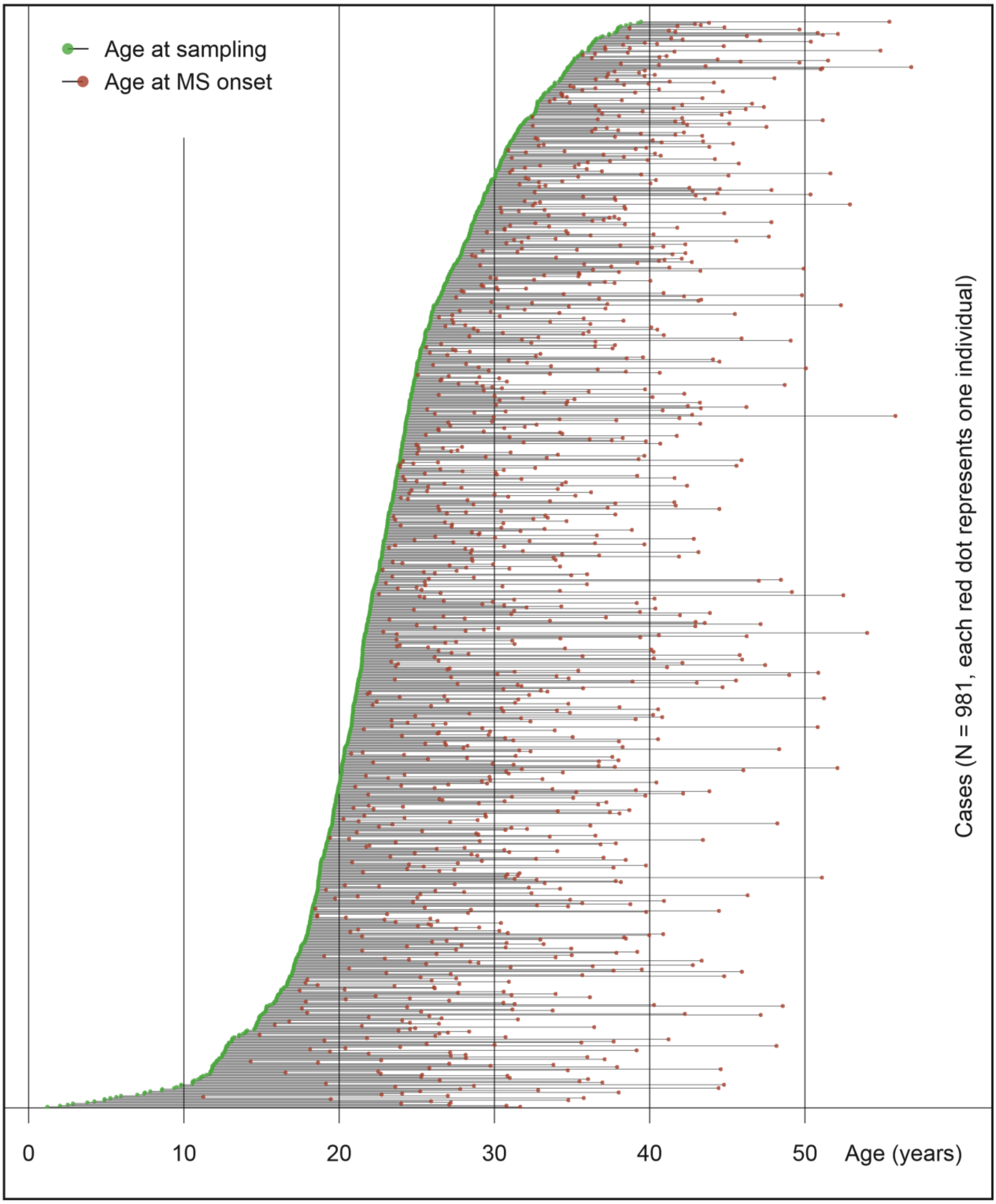

Supplementary Figure 2. Distribution of sampling by age and by clinical onset of MS. Cleveland dot plot of age at sampling relative to age at MS onset for each of the 981 cases.

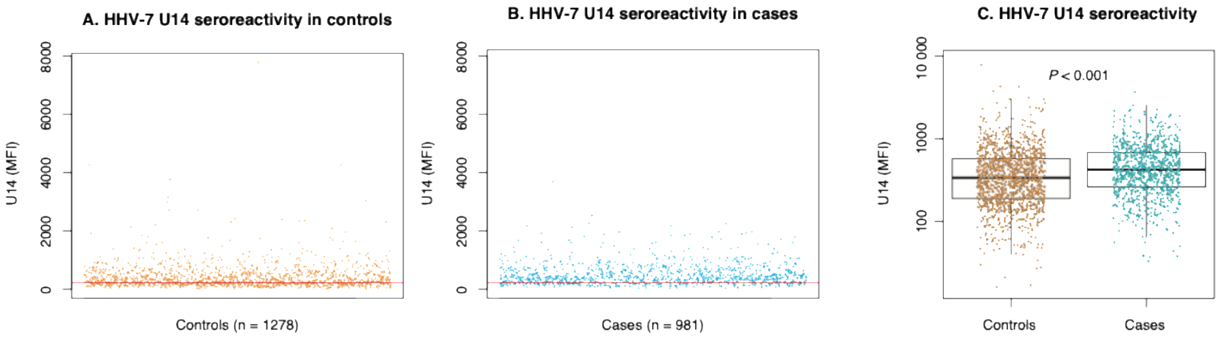

Supplementary Figure 3. Seroreactivity of HHV-7 antigen U14. (A) controls, (B) cases, and (C) in box plot comparison. HHV-7, human herpesvirus 7; MFI, median fluorescence intensity. Red lines in A and B represent cut-off at 225 MFI. In C, Mann-Whitney *U* test was used to compare seroreactivity between cases and controls.

Supplementary Table 2. HHV-7 seropositivity as a risk factor for developing MS. Univariate results divided by cohort and age at sampling.

2012 and 2020 cohort	Serostatus	OR	95% CI	P
All ages, n = 981 + 1278	HHV-7 +	2.19	1.76–2.72	<0.001
Age < 20, n = 291 + 431	HHV-7 +	2.34	1.62–3.36	<0.001
Age > 20, n = 690 + 847	HHV-7 +	2.12	1.61–2.78	<0.001
**2012 cohort**				
All ages, n = 628 + 628	HHV-7 +	2.09	1.56–2.81	<0.001
Age < 20, n = 122 + 122	HHV-7 +	2.43	1.30–4.53	<0.01
Age ≥ 20, n = 506 + 506	HHV-7 +	2.00	1.43–2.80	<0.001
**2020 cohort**				
All ages, n = 353 + 650	HHV-7 +	2.32	1.68–3.2	<0.001
Age < 20, n = 169 + 309	HHV-7 +	2.29	1.46–3.58	<0.001
Age < 20, n = 184 + 341	HHV-7 +	2.35	1.47–3.76	<0.001

Note: Number of cases and controls in each age strata; OR, Odds ratio; CI, Confidence interval; HHV-7 +, seropositive for Human herpesvirus 7; Cut-off for HHV-7 seropositivity is provided in Supplementary Table S1.

Supplementary Table 3. HHV-7 seropositivity as a risk factor for developing MS, adjusted for HLA genotype

All ages (n = 579 + 286)	OR	95% CI	*P*
HHV-7 +	1.59	(1.12–2.26)	<0.01
CMV +	0.75	(0.55–1.01)	0.06
EBV +	1.48	(0.95–2.31)	0.09
HHV-6A +	1.47	(1.05–2.06)	0.02
HLA-DRB1*15 +	2.94	(2.16–4.00)	<0.001
HLA-A*02 +	0.52	(0.38–0.70)	<0.001

Note: HLA data for cases was partially retrieved from previous Swedish studies (Immunomodulation and Multiple Sclerosis Epidemiology, Epidemiological Investigation of Multiple Sclerosis, and Genes and Environment in Multiple Sclerosis). Additional HLA data for controls and cases not included in the previous studies was retrieved from saliva samples. DNA was extracted and PCR analyses were performed as previously described in note 5 in the main manuscript (Ingvarsson et al, Eur J Neurol. 2024). Data on HLA-A*02 and HLA-DRB1*15 genotypes were thus available for 584 cases, and 307 and 313 controls, respectively. Only 176 of the cases appeared in sets where HLA data was also available for the matched controls, limiting the use of matched tests. Instead, we performed a logistic regression including all participants with HLA data. OR, Odds ratio; CI, Confidence interval; HHV-7 +, seropositive for Human herpesvirus 7; CMV +, seropositive for Cytomegalovirus; EBV +, seropositive for Epstein Barr virus; HHV-6A +, seropositive for Human herpesvirus 6A. Cut-offs for each viral antigen are provided in Supplementary Table S1.

Development and validation of a high through-put serological assay for HHV-7

Ethical Statement

This study was performed within the ethical standards of the Declaration of Helsinki.

All data included in this study were pseudonymized and the original donor identities could not be traced back. Therefore, no ethical permit was required according to the Regional Ethical Review Board in Stockholm, Sweden.

Serum samples

Serum samples from plasma donations were collected and aliquoted in four parallel aliquots per sample and kept at -70°C before analysis. One hundred individual samples were analyzed for serological response to the U14 protein of HHV-7. The analyses were done using a bead-based multiplex serology assay (hereafter referred to as bead based-assay) and a Luciferase Immunoprecipitation System (LIPS) assay, see specifications below.

Bead based assay

For serological IgG measurements for other antigens included in the panel, a multiplex serological assay using beads coated with recombinant glutathione s-transferase (GST) fusions proteins was used as described in detail elsewhere.[1,2] Briefly, the immunodominant viral protein U14 of HHV-7[3] (Full length; sequence ID: Q69495) was expressed as GST fusion protein using a modified pGEX vectors in *E. coli*. The antigen expressing bacteria were lysed, the lysate cleared of insoluble components and thereafter in situ purified on specific polystyrene beads set (SeroMap, Luminex Corporation) coupled to glutathione-casein (GC). Serum from the study subjects were diluted 1:1,000 and pre-incubated with GST lysate to remove antibodies specific for GST or wildtype bacterial proteins present in the lysate. Beads coated with different antigens were mixed and incubated with pre-incubated serum in filter bottom 96-well plates. After washing, a biotinylated goat-anti human IgG secondary antibody (Dianova) was added and, after additional washing steps, detected by streptavidin-R-phycoerythrin (Moss). Median fluorescence intensity (MFI) was measured with a Luminex 200 analyzer. On every plate, four plate controls were tested to assess assay variation, as described in detail elsewhere,[4,5] yielding median coefficients of variation for other antigens included in the panel of 8–17%. The plate controls did not react with the HHV-7 antigens.

LIPS assay

The Luciferase ImmunoPrecipitation assay System has been described previously.[6,7] In brief, genes of interest are cloned in frame with a FLAG-tagged renilla luciferase gene using the pREN2 vector. All constructs were verified by restriction enzyme profiling and DNA sequencing. The entire HHV-7 U14 gene (Full length; sequence ID: Q69495) was PCR amplified using Q5 Hi-Fi DNA polymerase using HHV-7 infected Supt1 cells as source of HHV-7 DNA. The PCR fragment was cloned into the BamH1-Xho1 digested pREN2 vector by Gibson assembly cloning. The pREN2-GST negative control vector was generated as described.[8] Protein expression was validated by western blot using anti-FLAG antibodies. To prepare lysates for the LIPS assay, HEK293T cells seeded the day before at 4 x10^6 cells/10 cm^2 petri dish, were transfected with 8 µg of vectors using PEI. Forty-eight hours post-transfection, cells were harvested and lysed as previously described.[6,7] Anti-Flag antibodies were used as positive control. Each serum was tested in duplicate at a final dilution of 1:100. Sera were incubated with the lysate containing 10^6 relative luciferase unit (RLU) of the target antigen in wells of a 96-well plate for 2h at room temperature with shaking (300 rpm) after which protein A coated magnetic spheres were added to the wells with moderate shaking (300 rpm). After 60 minutes, the plates were loaded onto an automatic ELSA plate washer equipped with a magnetic stand. After 3 washes, the buffer was removed, and the plates loaded into a luminometer with an automatic substrate dispenser (TECAN M200 reader, Morrisville, NC, USA). Light emission was measured over 10 seconds with a 2 sec start delay.

Statistical calculation

Linear regression analysis was performed using the computer program Microsoft Excel.

Results

Paired aliquots from one hundred paired serum samples were analyzed with a bead-based and a LIPS assay as described above. The two assays gave similar results as seen by linear regression analysis (R^2 = 0.7249; p=3.2e-29) (**Figure S4**).

A B

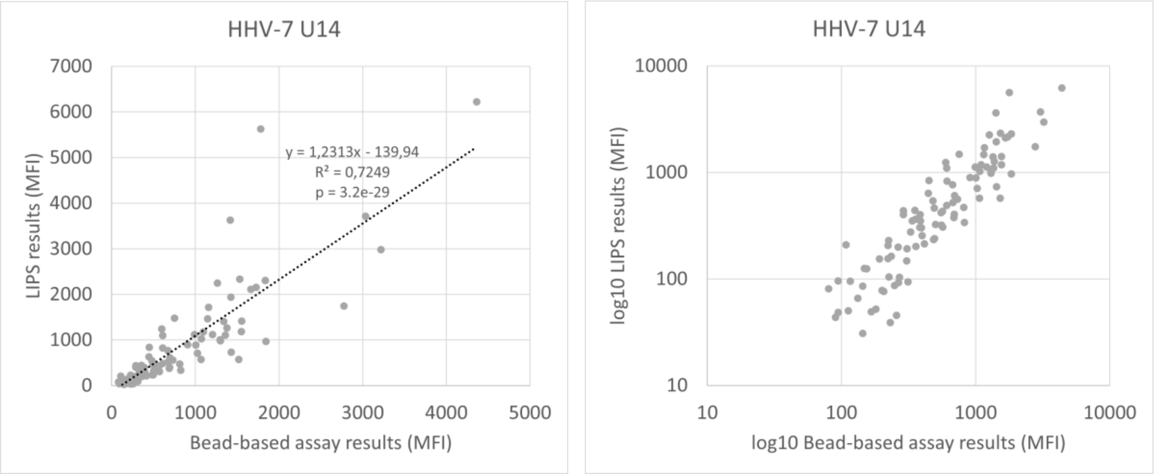

Supplementary Figure 4. Serological measurements using the LIPS and bead-based assays on paired serum samples from one hundred individuals. Data were plotted (A) linear or (B) log10 transformed.

References

1. Waterboer T, Sehr P, Michael KM, et al. Multiplex human papillomavirus serology based on in situ-purified glutathione S-transferase fusion proteins. *Clin Chem*. 2005;51(10):1845-1853. doi:10.1373/CLINCHEM.2005.052381,
2. Waterboer T, Sehr P, Pawlita M. Suppression of non-specific binding in serological Luminex assays. *J Immunol Methods*. 2006;309(1-2):200-204. doi:10.1016/j.jim.2005.11.008
3. Stefan A, De Lillo M, Frascaroli G, Secchiero P, Neipel F, Campadelli-Fiume G. Development of Recombinant Diagnostic Reagents Based on pp85(U14) and p86(U11) Proteins To Detect the Human Immune Response to Human Herpesvirus 7 Infection. *J Clin Microbiol*. 1999;37(12):3980. doi:10.1128/JCM.37.12.3980-3985.1999
4. Kreimer AR, Johansson M, Yanik EL, et al. Kinetics of the Human Papillomavirus Type 16 E6 Antibody Response Prior to Oropharyngeal Cancer. *J Natl Cancer Inst*. 2017;109(8). doi:10.1093/JNCI/DJX005,
5. Michael KM, Waterboer T, Sehr P, et al. Seroprevalence of 34 Human Papillomavirus Types in the German General Population. *PLoS Pathog*. 2008;4(6):e1000091. doi:10.1371/JOURNAL.PPAT.1000091
6. Burbelo PD, Bren KE, Ching KH, et al. LIPS arrays for simultaneous detection of antibodies against partial and whole proteomes of HCV, HIV and EBV. *Mol Biosyst*. 2011;7(5):1453-1462. doi:10.1039/C0MB00342E

7. Burbelo PD, Ching KH, Klimavicz CM, Iadarola MJ. Antibody Profiling by Luciferase Immunoprecipitation Systems (LIPS). *J Vis Exp*. 2009;(32):1549. doi:10.3791/1549

8. Peddu V, Dubuc I, Gravel A, et al. Inherited Chromosomally Integrated Human Herpesvirus 6 Demonstrates Tissue-Specific RNA Expression In Vivo That Correlates with an Increased Antibody Immune Response . *J Virol*. 2019;94(1). doi:10.1128/JVI.01418-19,
